# Perioperative risk factors impact on intensive care unit length of stay (ICU length of stay) in oral squamous cell carcinoma

**DOI:** 10.1186/s12903-023-03304-4

**Published:** 2023-09-01

**Authors:** Abdo Ahmed Saleh Mohamed, Lianxi Mai, Guangxin Rao, Song Fan, Mubarak Ahmed Mashrah, Mohamed Ali Mahyoub Holkom, Chaobin Pan, Zhouyu Lin

**Affiliations:** 1 Department of Oral & Maxillofacial Surgery, Sun Yat-sen Memorial Hospital, Sun Yat-sen University, 107 Yanjiang Road, Guangzhou, 510120 China; 2 Department of Oral and Maxillofacial Surgery, Faculty of Dentistry, Ibb University, Ibb, Yemen; 3 Department of Oral Implant, Guangdong Engineering Research of Oral Restoration and reconstruction, Guangzhou Key Laboratory of Basic and Applied Research of Oral Regenerative Medicine, Affiliated Stomatology Hospital of Guangzhou Medical University, Guangzhou, Guangdong China; 4 Department of Oral & Maxillofacial Surgery- head & neck oncology, School of Stomatology, Wuhan University, Wuhan, Hubei China

**Keywords:** Oral squamous cell carcinoma, Risk factor, ICU admission, Microvascular free flap

## Abstract

**Background:**

The trend in postoperative care for free flap patients is to deescalate from routine ICU admission into a specialty recovery unit. This study aims to investigate the predictive parameters in a routine perioperative clinical assessment that are expected to be directly correlated with prolonged ICU length of stay in at-risk patients who received oral reconstructive surgery for squamous cell carcinoma (OSCC).

**Methods:**

All patients who underwent ablative surgery for OSCC with free flap reconstruction and were managed in the ICU were included in this study. The primary outcome was ICU-length of stay. Perioperative, operative and postoperative parameters were analyzed using single test ( *t*-test, ANOVA analysis, correlation coefficients, effect size) and multivariate regression test. The *P*-value was set as < 0.005 to be considered statically significant.

**Results:**

The study included 136 homogeneous patients, with a mean ICU length of stay of 4.5 (± 4.43 day). Patients with pre-operative positive renal dysfunction (P = 0.004), peripheral vascular disease (P < 0.001), postoperative complications (P = 0.028) or positive heart failure class III (P < 0.001 ) were recognized as at-risk patients for a significantly longer ICU length of stay.

**Conclusion:**

Patients with perioperative severe renal dysfunction, peripheral vascular disease, postoperative complication or high NYHA class are prone to have a significantly longer ICU length of stay. Several factors were considered as confounders contributing to increased ICU management time in combination with other variables. Additionally, in highly risk patient, the presence of the highly trained medical support, including the appropriate nursing care, is more critical than those patients without these risk factors.

## Introduction

Oral cancer is a global health concern, with 409,000 cases and 135,000 deaths reported in 2013 [1]. Smokeless tobacco, cigarettes, alcohol, and HIV infections with human papilloma virus (HPV, type 16 and 18), are considered the major risks factors for oral cancer [2, 3]. Oral cavity cancer is found to be more frequent in women aged between 50 and 70 years, and among men aged between 55 and 65 years. The most common form of the oral cavity malignant tumor is the oral squamous cell carcinoma (OSCC), accounting for 90% of all oral cancer cases [4–6]. Microvascular free flap (MVFF) is the primary treatment option for OSCC, with a reported survival rate of 90–95% [7]. However, OSCC surgery with MVFF can be invasive, complex, and demanding, often requiring postoperative ICU admission for long-term mechanical ventilation, cardiopulmonary monitoring, or sedation [6, 8, 9]. Recently, there has been a trend toward deescalating postoperative care for patients underwent MVFF, moving them from routine ICU admission to specialized recovery units. However, the rate of ICU admission remains highly variable between hospitals and even within the same clinical center [10, 11]. Some studies suggest that patients undergoing complex OSCC surgeries can be safely admitted to non-ICU wards without complication [12]. A meta-analysis concluded that the direct admission to ICU after head and neck microvascular reconstructive operations did not reduce the incidence of complication rates or flap failure [13]. However, the majority of reconstruction surgeons still prefer to admit their patients to the ICU immediately after surgery [11, 14].

The length of stay (LOS) in the ICU, from admission to discharge or transfer to a step-down unit, is a medically and economically relevant factor for both the clinical center and patient [10, 14–16]. Several studies have focused on the benefits of ICU admission versus non-ICU admission for microvascular reconstructed flap patients, including postoperative complication, mortality rate, flap success rate, and postoperative delirium [9, 11, 17–23]. Prolonged ICU length of stay can trigger several problems such as infection and thromboembolic events. Furthermore, prolonged ICU length of stay can further mask clinical symptoms and therefore humper critical diagnoses such as cerebral insult [24].

However, little research has been done on the effect of perioperative risk factors on the length of stay in the ICU for OSCC patients.

To address this gap, this study aims to investigate the routine perioperative clinical assessment to identify predictive parameters that may be correlated with prolonged ICU length of stay in patients undergoing OSCC surgery with free flap reconstruction. The null hypothesis of this study is that there is no significant direct relationship between postoperative ICU length of stay and the predicted parameters.

## Materials and methods

### Study design

This study was conducted with the guidelines of The Helsinki Declaration and received approval from the Ethics committee of the Memorial Hospital of Sun Yat-sen University (Ethical Approval Number: SYSKY-2023-027-01) All patients were treated at the memorial Hospital of Sun Yat-sen University Guangzhou, China, and informed consent was obtained from all participants prior to the treatment. Data collection and analysis were carried out by a resident in oral and maxillofacial surgery.

A retrospective electronic clinical chart review was conducted over a five-year period (2017–2021). The protocols for surgery, anesthesia, and laboratory were collected based on specific inclusion and exclusion criteria. Patients were eligible for inclusion if they met the following criteria: underwent ICU admission directly after surgery, aged between 18 and 80 years, had biopsy-proven OSCC, underwent neck dissection with compartment surgical resection of the tumor, and received free flap reconstruction with a surgical time of at least 5 h. Patients were excluded from the study if they were under 18 or over 80 years of age, had a benign tumor, underwent trauma defect reconstruction or received postoperative management in a step-down unit, intermediate care unit, or other non-ICU specialty ward. Additionally, patients who received other forms of defect reconstruction, such as temporary/permanent obturator or regional/ local flap reconstruction, were excluded from the study.

A perioperative risk factor assessment was performed for diabetes mellitus, coronary artery disease, chronic alcoholism, renal dysfunction, chronic smoking, peripheral vascular disease (PVD), and chronic obstructive pulmonary disease (COPD). Demographic and surgical data were analyzed, including age, sex, primary tumor location/site, body mass index, American Society of Anesthesiology (ASA), heart failure, tumor classification (TNM), and operation time, and were compared to ICU length of stay. The amount of intraoperative blood transfusion, hemoglobin level and urinary output were also analyzed. The postoperative complications (Clavien-Dindo classification [25] ) and ICU length of stay analysis were conducted.

### Sample size

One hundred thirty-six patients who underwent oral and maxillofacial reconstruction and were admitted to the ICU between 2017 and 2021 were considered as the sample size.

### Surgical procedure

All patients in this study underwent a compartment surgical resection of the OSCC tumor. A compartment resection implies that all tumors were resected en-bloc with safe margin more than 1 cm. An intra-operative microscopic analysis (frozen biopsy) was performed to ensure the tumor-free margins. The neck dissection was performed based on the T-stage and in accordance with the guidelines of the International Cancer Committee. The study included patients who underwent microvascular free flap reconstruction.

In our practice, most patients were admitted to the recovery room for 1–3 h and then transferred to the surgical ward. The ICU management for the immediate postoperative period is indicated for patients with specific conditions. All surgeries were performed at the Oral and Maxillofacial department of Memorial Hospital, Sun Yat-sen University, Guangzhou, China, with patients admitted to the ICU for immediate postoperative care.

Patients were admitted to the ICU immediately after the surgery if they had severe organ functional impairment, risk factors, and comorbidity. In some cases, admission to the ICU was due to the absence of appropriate step-down units or the decision of the operating surgeon. However, the length of stay in the ICU for these patients was not more than 24 h. The reasons and criteria for ICU admission postoperatively have been reported in various studies [10, 12, 22, 26–28]. Patients were admitted to closed ICU based on their co-morbidities, risk factors, ASA score, and intra-operative health condition [10, 19, 22]. The closed unit was staffed by highly trained intensive-care physicians and was designed to provide specialized management for admitted patients.

### Postoperative patient management

In the postoperative period, patients receive comprehensive care including appropriate staffing ratios, medical support, wound management, and flap monitoring. Special attention was given to maintaining the patient’s hemodynamic stability in the postoperative period. Mechanical ventilation and sedation were utilized based on various factors such as postoperative cardio-pulmonary function, presence of postoperative delirium or others conditions [10, 17, 20, 23]. Our efforts to minimize the rate of postoperative delirium include administering medications, including narcotics, based on established anesthesia standards and protocols. All ICU staff were trained in caring for post operation cancer patient and monitoring free flaps. The nurse-to-patient ratio during this study was 1:3 and postoperative flap monitoring was performed every hour during the first 24 h, and followed by monitoring every 6 h until the 6th day post-surgery.

During the ventilation period, patients were administered Propofol and Remifentanil. All patients received nutrition through a nasogastric tube for the first five days after surgery. Ventilator wean was conducted for all patients on the first postoperative day. The sedation was stopped in patients who no longer required inotropic support, were able to maintain spontaneously ventilation and gas exchange, had hemodynamic stability and had a normal average blood PH. The decision to discharge patients from the ICU was based on their condition and the agreement between the anesthesiologist and oral and maxillofacial surgeon.

### Statistical analysis

The primary outcome parameter was ICU length of stay in hours. The demographic and perioperative variables were analyzed to see their correlation with ICU length of stay. Additionally, the number of intra-operative and postoperative complications was also considered. Some variables such as intra-operative blood loss, hemoglobin level, primary tumor size and urinary output were analyzed using descriptive statistics.

To evaluate the relationships between the variables and the primary outcome, a mean analysis was conducted on dichotomous, numerical, and categorical variables using t-test, Mann Whitney U test, and ANOVA, as appropriate. The differences between the primary outcome and the parameters were analyzed using the Correlation coefficient (Pearson and Spearman). The effect size was calculated using Cohen’s d for t-test and Eta Square n^2^ for ANOVA test. However, it is important to acknowledge that single tests only focus on the effect of a single parameter, without considering the influence of other co-variables that may also affect ICU length of stay in parallel.

To better understand the correlation between the various variables and the primary outcome, both univariate and multivariate linear regression models were utilized. The multivariate linear regression model was performed to examine the unadjusted associations between the ICU length of stay and potential risk variables. The R^2^ value was calculated to determine the correlation between the observed and predicted values and to assess the validity of the regression model.

A 95% confidence intervals were given for *t*-test and a p-value *p* < 0.05 was considered statistically significant. All descriptive statistics and calculations were presented as the mean ± standard deviations (SD), unless otherwise noted. All statistics analysis in this study was performed using SPSS (V26) and the figures were generated by SPSS and GraphPad software (9.1.4; Inc., USA).

## Results

**Fig. 1:**
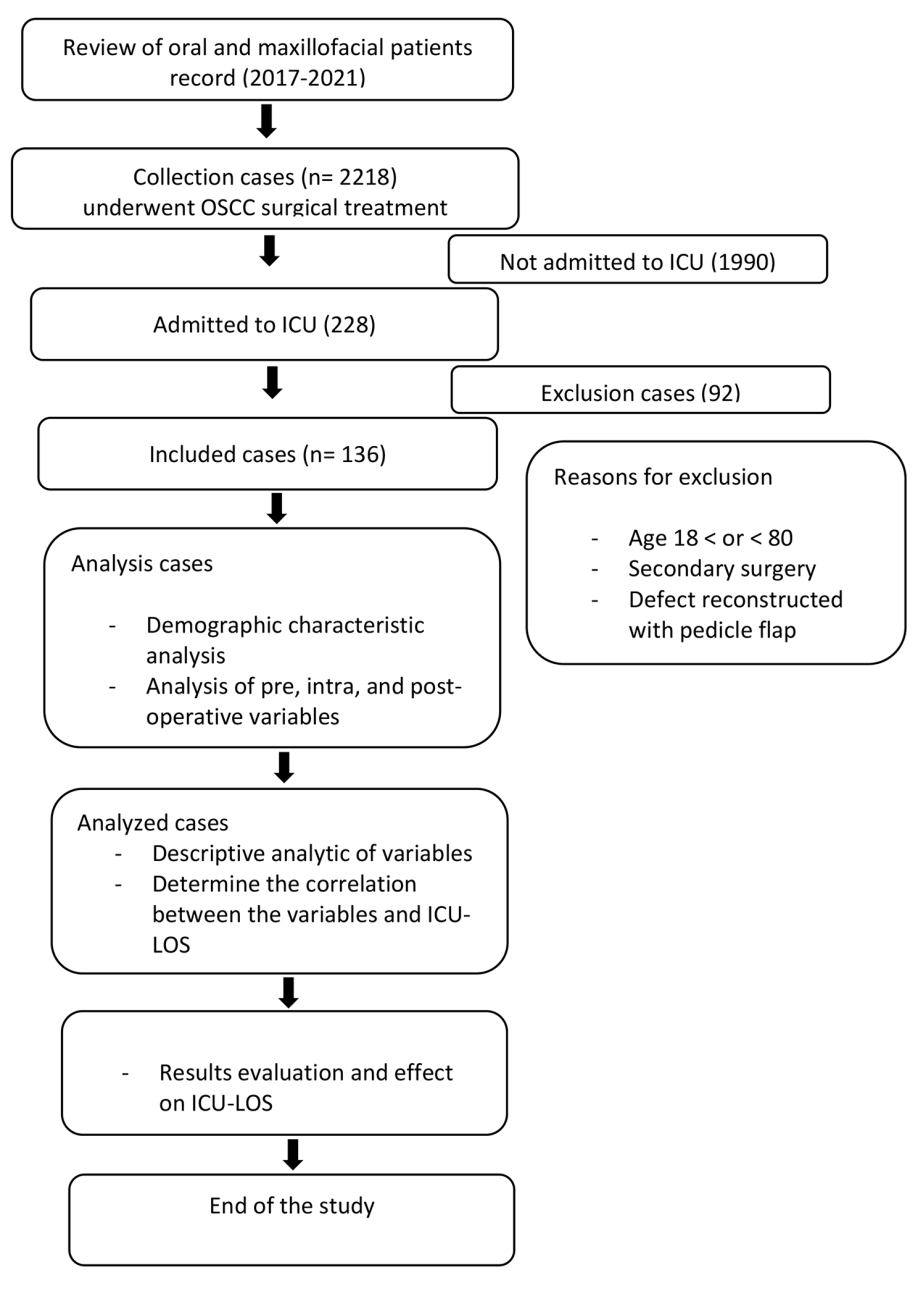
The study protocol

During the recruitment period of this study, a total of 2218 patients were treated for oral and maxillofacial squamous cell carcinoma, of whom 229 patients (10.3%) were requiring immediate ICU admission. Of these, 136 patients met the inclusion criteria and were selected for this study (as shown in Fig. 1). All included patients underwent surgical resection and reconstruction with microvascular free flaps. The study population had a mean age of 67.9 ± 13.6 years, with 64 (47.1%) being female. The tongue was the most common primary site, accounting for 56 cases (40.6%), followed by the floor of the mouth (29 cases, 21%), the cheek (19 cases, 13%), and gingiva and oropharynx (for each 9 cases, 6.5%) (Table 1). Most postoperative complications were managed non-surgically using pharmacological intervention, except for five patients (3.6%), who required reoperation for anastomosis revision, or stop of bleeding. Complete flap loss occurred in three patients 2.2%, and second-free flap was harvested immediately. There were no other complications during ICU. length of stay, or at ICU discharge. One patient died during hospitalization because of the heart failure.

**Table 1:** Demographic data

Gender	No. (%)	Mean of age (SD)
Male	72 ( 52.9)	66.39 (14)
Female	64 (47.1)	79.78 (13)
Over all	136	67.9 ± 13.6
Tongue	56 (40.6%)	
Floor of the mouth	29 (21%)	
Cheek	19 (13.8%)	
Gingiva	9 (6.5%)	
Oropharynx	9 (6.5%)	
Mandible	8 (5.8%)	
Maxilla	6 (4.4%)	

**Fig. 2:**
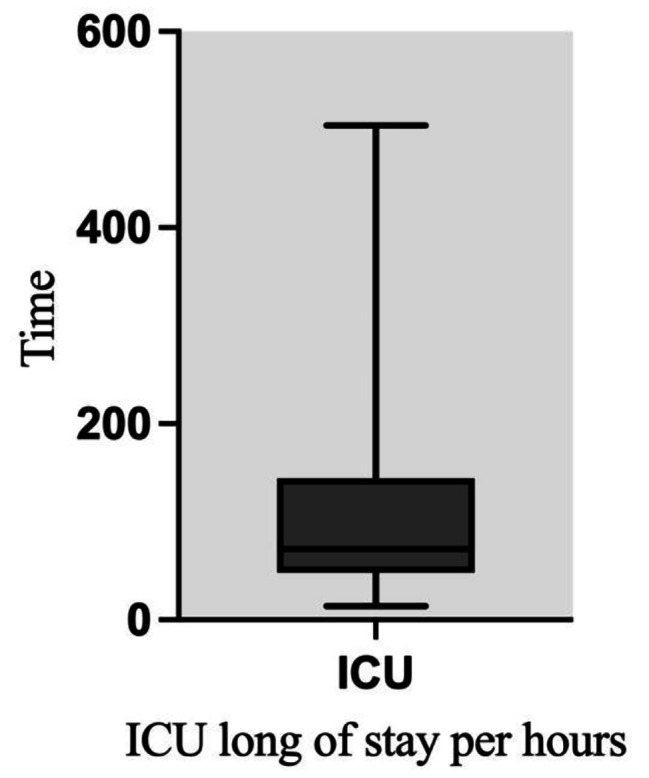
The distribution of primary outcome (ICU-length of stay) for 136 patients

**Fig. 3:**
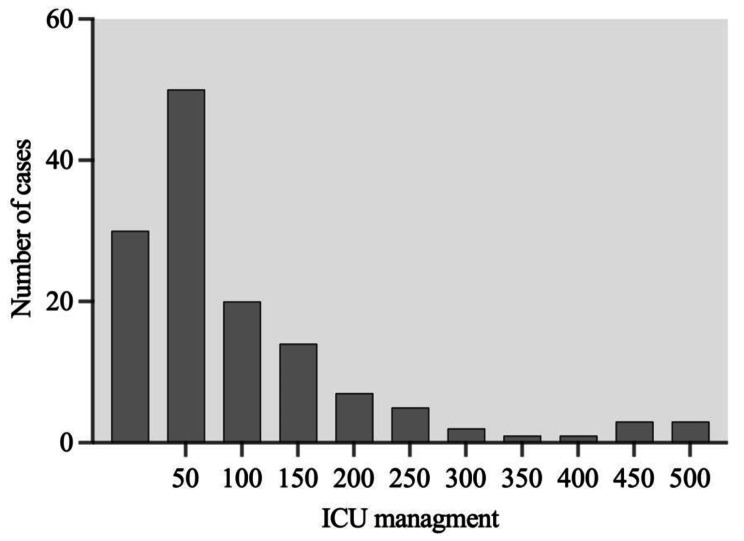
Frequency distribution of patient admitted to ICU management

The parameters of hemoglobin, albumin, neutrophil, intra-operative bleeding, urinary output and intra operative fluid were summarized in Table 2. The mean of ICU length of stay was 108.24 ± 106.48 h (Fig. 2; Table 3). The distribution of patients admitted into ICU was also illustrated in Fig. 3. There was no significant difference between male (103.64 ± 94.7 h) and female (113.42 ± 118.8 h). The mean operative time was 7.49 ± 1.6 h. The single tests revealed a highly positive correlation coefficient or significant with highly effect sizes ( Cohen’s d or Eta Square η2 ) (Table 4).

**Table 2:** Intra-operative parameters

Intra-operative parameter	Min	Max	Median	Mean	SD
Operative time (h)	5	13	7	7.49	1.6
Hemoglobin level g/L	67	178	122	121.32	22.48
Albumin (g/L)	20	81	36.65	36.92	7.22
Platelet (10^9/L)	57	654	239	256.4	93.95
Neutrophil (10^9/L)	0.80	36	4.46	5.14	3.70
Lymphocyte (10^9/L)	0.19	4.10	1.45	1.52	0.63
Intraoperative bleeding (mL)	220	840	300	342.30	118.69
Urinary output (mL)	350	2250	600	755.92	437.25
Intra operative fluid (mL)	740	6500	2650	2813	959.76

**Table 3:** Primary outcome descriptive analysis

	Min	Max	Median	Mean	SD
ICU length of stay (h)	14	504	72	108.24	106.48
ICU length of stay (d)	0.58	21	3	4.5	4.43

**Table 4:** Comparing between means in different variables

Para meter	Testing	P-value	Effect size
Gender	*t*-test	0.595	Cohens’d = 0.17(0.09)
Arterial hypertension	Mann-Whitney U test	0.001	η^2^ = (017)
Debits mellitus	*t*-test	0.427	Cohens’d = 0.19
CAD	Mann-Whitney U test	0.044	η^2^ = 0.03
Renal dysfunction	*t*-test	< 0.001	Cohens’d = 1.61
Alcoholism	*t*-test	0.151	Cohens’d = 0.37
Smoking	*t*-test	0.758	Cohens’d = 0.12
COPD	Mann-Whitney U test	0.001	η^2^ = 0.07
ASA	ANOVA	< 0.001	η^2^ = 0.27
PVD	*t*-test	< 0.001	Cohen’s d = 2.04
Extent heart failure	ANOVA	< 0.001	η^2^ = 0.62
Postoperative complications	ANOVA	< 0.001	η^2^ = 0.503

The correlation between the variables and the primary outcome was tested (Table 5). The variables that were significantly correlated with ICU length of stay or p value was less than 0.1 was subjected to multivariate regression test.

**Table 5:** Correlation between the different variables and ICU length of stay

Parameter	Test	Correlation Coefficient	P value
Gender	Pearson test	0.046	0.595
Age	Spearman’s test	R = 0.187	0.030
BMI	Spearman’s test	R = 0.181	0.036
Operative time	Spearman’s test	R = 0.277	0.001
Blood transfusion	Spearman’s test	R = 0.228	0.008
Chronic smoking	Pearson test	0.027	0.758
Chronic alcoholism	Pearson test	0.168	0.052
Tumor classification	Spearman’s test	0.206	0.016
Artery hypertension	Spearman’s test	0.419	< 0.001
Diabetic mellitus	Pearson test	0.069	0.427
Coronal artery disease (CAD)	Spearman’s test	0.174	0.043
Nick dissection	Pearson test	0.261	0.002
Renal dysfunction	Pearson test	0.554	< 0.001
Chronic Obstruction Pulmonary Disease (COPD)	Spearman’s test	0.275	0.001
Peripheral vascular disease	Pearson test	0.755	0.001
Heart Failure	Spearman’s test	0.842	< 0.001
ASA	Spearman’s test	0.663	< 0.000
Location of the tumor	Spearman’s test	0.011	0.903
Hemoglobin	Spearman’s test	-0.136	0.119
Platelet	Pearson test	0.065	0.454
Albumin	Pearson test	-0.178	0.039
Neutrophil	Pearson test	0.029	0.737
Lymphocyte	Pearson test	0.144	0.096
Urinary output	Pearson test	0.061	0.484
Intra-operative fluid	Pearson test	0.110	0.207
Postoperative complication	Pearson test	0.6390.639	0.001

**Fig. 4:**
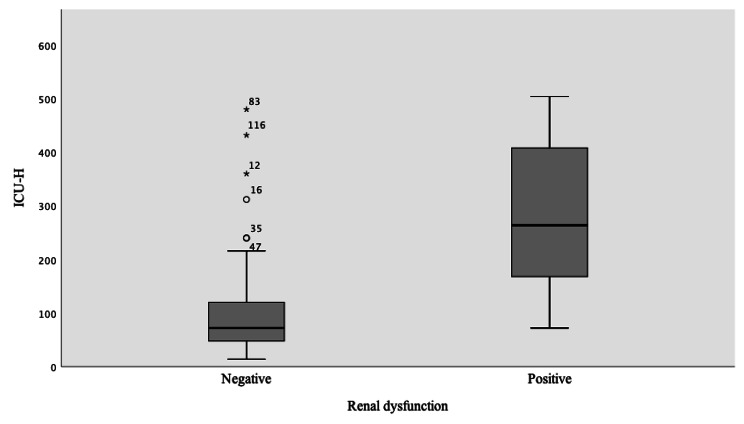
ICU patients with Renal dysfunction disease

**Fig. 5:**
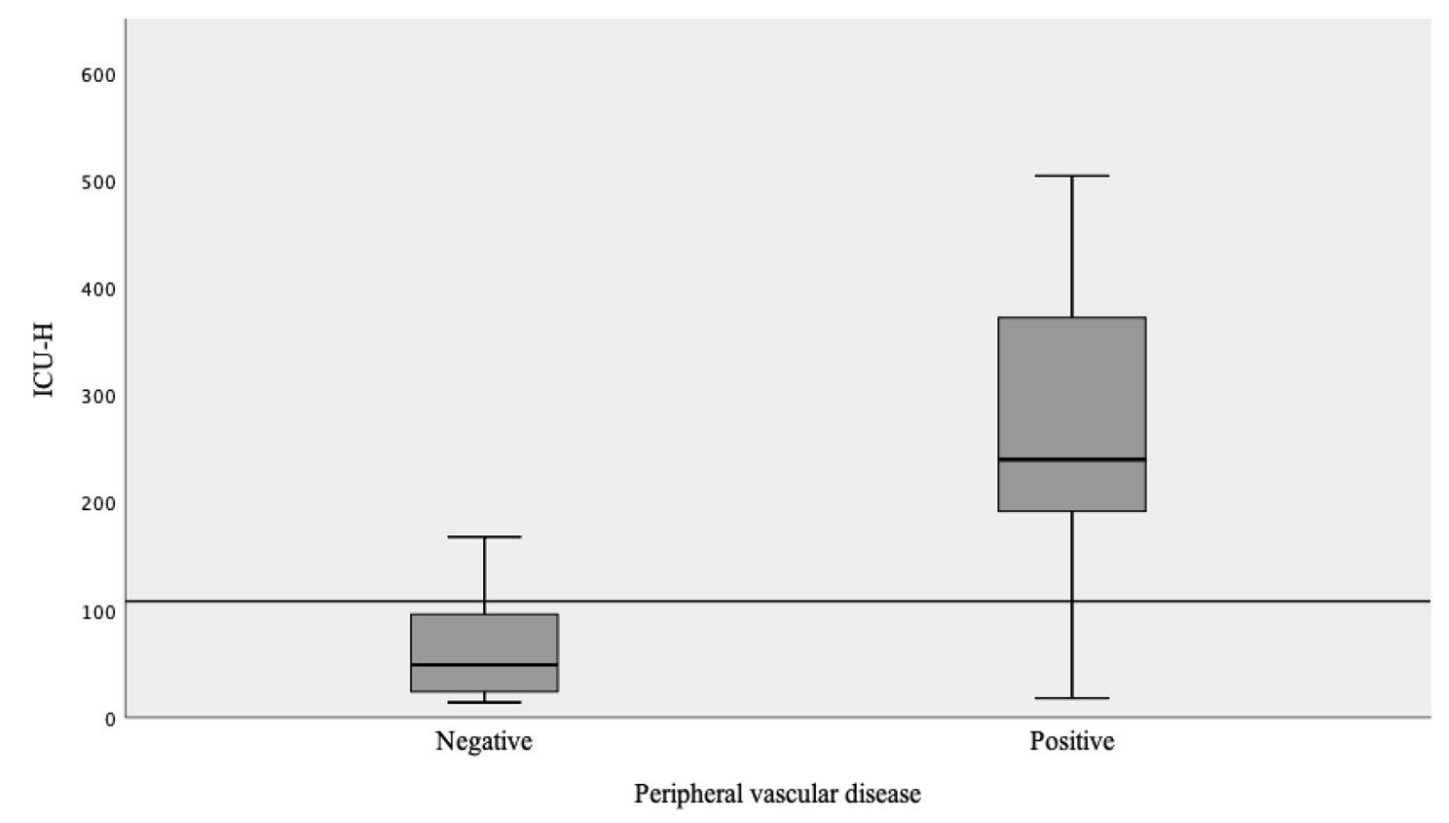
ICU patients with the peripheral vascular disease

**Fig. 6:**
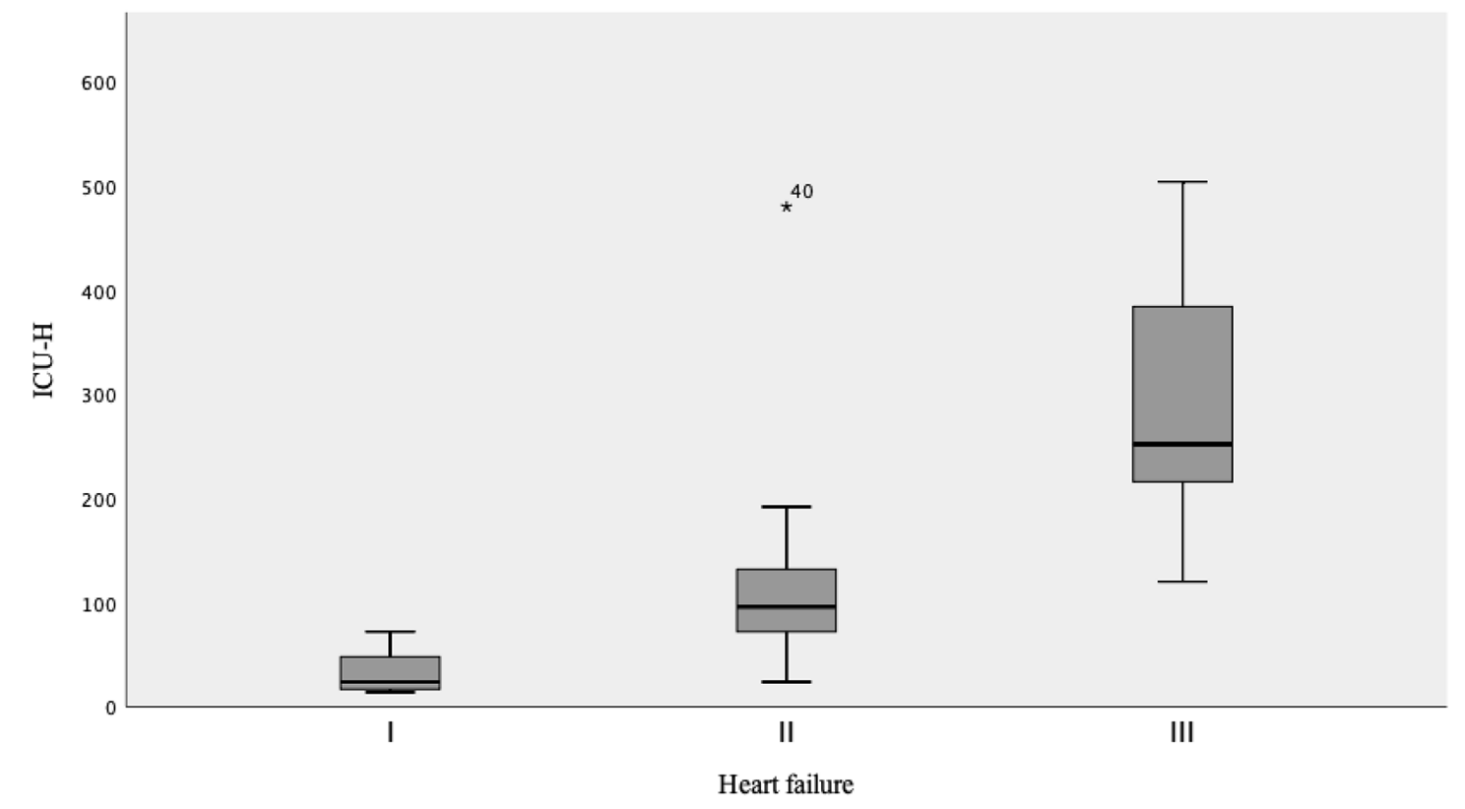
ICU patients with heart failure disease

The increase in grade of T-classification of tumor size, ASA, and the need of the blood transfusion was found to be correlated with an increase in ICU length of stay (Table 5). The univariate general linear regression model showed significant value (p < 0.05) for the variables; renal dysfunction, ASA III, heart failure, COPD, PVD, postoperative complication, and neck dissection (Table 6). Renal dysfunction (p = 0.004), PVD and Heart failure, showed the strongest correlation with ICU length of stay (P < 0.001, for both) (Figs. 4, 5 and 6). The average of ICU length of stay increased by 75.8% in patients with positive renal dysfunction, 79.7% in cases with PVD, 66.8% in patients with heart failure class III, and 72% in postoperative compilations. The regression model had a quality value of R^2^ = 0.77 indicating that 77% of the patient cohort had a valid of estimate of the correlation (Table 7).

**Table 6:** Unilinear regression model

Parameter	Min		Max		Mean(SD)	R^2^	P Value
Age	24		93		67.99 (13.6)	0.025	0.065
BMI	19		88		56.94 (12)	0.17	0.128
Time of operation (H)	5		13		7.49 (1.6)	0.016	0.139
Parameter		n		Mean ICU-LOS (SD)			P-Value
Sex	Male	72		103.6(94.7)		0.002	0.595
	Female	64		113.42(118.8)			
ASA	I	14		30.6 (24)		0.253	0.000
	II	34		44.2 (23.8)			
	III	75		133 (113.3)			
	IV	13		216 (105.5)			
Tumor classification	T1	24		135.1 (178.3)		0.001	0.705
	T2	43		78.93(59)			
	T3	41		110.8(86.9)			
	T4	28		126.39 (103)			
Blood transfusion	0 unit	42		77.76(88.9)		0.018	0.118
	One unit	19		148.5(147.4)			
	Two unit	30		103.2(76.07)			
	Three unit	29		132.53(24.6)			
	> three unit	16		124.6(70.6)			
Arterial Hypertension	Positive	44		130.18 (66.2)		0.020	0.97
	Negative	92		97.75 (120)			
Diabetes Mellitus	Positive	22		124.8 (98.11)		0.005	0.427
	Negative	114		105.04 (108.1)			
Neck dissection	Unilateral	113		95.77(99.3)		0.068	0.002
	Bilateral	23		169.51(120.7)			
CAD	Positive	18		74.33 (80.7)		0.16	0.148
	Negative	118		113.4 (109.24)			
Renal dysfunction	Positive	15		275.2 (143.6)		0.307	< 0.001
	Negative	121		87.55 (80.2)			
Chronic alcoholism	Positive	24		146.9 (148.8)		0.028	0.052
	Negative	111		100.2 (94.3)			
Chronic smoking	Positive	39		110.3 (107.5)		0.001	0.758
	Negative	96		104 (106.2)			
COPD	Positive	34		144.7 (127.4)		0.039	0.021
	Negative	102		96.09 (96.18)			
PVD	Positive	28		265.5 (129.89)		0.570	< 0.001
	Negative	108		67.47 (43.5)			
Heart failure	I	50		33.3 (18.07)		0.567	< 0.001
	II	64		106.8 (64.55)			
	III	22		282.5(120.09)			
Postoperative complication	Stage 0	65		42.91(26.4)		0.567	0.001
	Stage I	30		104.9 (74.2)			
	Stage II	31		229.9(130.6)			
	Stage III	10		165.6(70.1)			
	Stage IV	0		0			

**Table 7:** Multivariate regression model

Parameters	R^2^ = 0.77		
	Coefficient Std.	Standardized Coefficient Beta	P value
Age	0.347	0.067	0.137
BMI	0.433	0.003	0.951
Operative Time	2.868	0.066	0.151
Blood transfusion	3.566	0.022	0.642
Chronic alcoholism	12.392	0.088	0.051
Tumor classification	4.919	0.073	0.116
Artery hypertension	11.576	0.041	0.425
Nick dissection	13.872	0.019	0.693
Coronal arterial disease	14.171	0.065	0.157
Renal dysfunction	19.46	0.163	0.004
Chronic Obstructive Pulmonary disease	11.831	0.036	0.478
Peripheral vesicular disease	16.908	0.346	< 0.001
Heart Failure	10.877	0.329	< 0.001
ASA	7.779	0.061	0.286
Postoperative complication	6.936	0.144	0.028

## Discussion

Free flaps reconstruction for advanced head and neck cancer often requires admission to the ICU or an intermediate care unit [11]. It is important to highlight that, postoperative ICU admission is not a standard practice in many centers. A recent meta-analysis revealed that immediate postoperative ICU admission after head and neck microvascular reconstruction surgery did not decrease the flap failure rate or complications, and concluded that routine admission to the ICU should be limited to specific patients [13]. In literature, papers have frequently been discussed the prognostic parameters for a longer ICU length of stay [9, 10, 19, 29]. Therefore, this study did not aim to question the recommendations or guidelines patient care in head and neck microvascular surgery. Even though suitable postoperative patient management varies widely between centers [10], there is also a lack of consensus on specific criteria for determining the need for postoperative ICU versus non-ICU admission for at-risk patients [22]. This study is the first to investigate factors that may directly affect ICU length of stay in high-risk patients with OSCC.

In general, perioperative comorbid status, interoperative complication, surgeon’s preference and to some extent, the financial and human resources of the clinical center determine the need of ICU admission [10, 18, 22, 29, 30]. Referring to the postoperative care in major head and neck cancer patients, it was suggested that patients with perioperative comorbidities, cardio-pulmonary impairment, high ASA grade, or other risk factors should be considered for admission into ICU [12, 26, 28].

In this cohort, many patients with above-stated conditions were present. Additionally, blood transfusion, albumin level, treatment of postoperative complication and other perioperative comorbidities were observed. Many studies suggest the ICU admission as the appropriate care for cases with previously mentioned comorbidities [12, 26, 28].

ICU length of stay is a significant medical and financial factor associated with an increased risk of pneumonia, narcotic use, higher hospital charges, and increased staffing requirements [9, 10, 19, 31]. The goal of this study was to identify at-risk patients based on their perioperative status and comorbidities, and predict which patients are likely to require a prolonged critical care management phase.

In the study cohorts, the tongue was the most common primary tumor site, affecting 40% of patients. In a multicenter study of oral cancer, Dhanuthai et al. [32], likewise described a similar finding of cancer distribution. Furthermore, our finding regarding the age, urinary output, and blood loss during surgery was comparable to other studies’ cohorts [19, 20].

The decision of the admission was taken preoperatively in most cases based on age, BMI, ASA, COPD, heart disease, renal dysfunction, and extension of the tumor. However, the intraoperative decision was made for some cases with intraoperative parameters such as intra operative blood transfusion, arterial hypertension, availability of appropriate step-down units, or by the decision of the surgeon.

The average ICU length of stay in this study was 108.24 h.

(4.5 days), which is longer compared to other published studies where the majority of ICU length of stay ranged between 24 and 72 h [7, 9, 14, 33–35]. The longer ICU length of stay in our study could be due to the higher risk profile of our patients, who had underlying conditions such as heart disease, pulmonary disease, and renal dysfunction. The mean of surgery time was 7.4 h which is similar to that reported in other published papers in this field [19, 20, 29].

Although parameters such as IBM, patient age, operative time, tumor classification, coronal artery disease, hypertension, neck dissection, COPD and albumin showed significance in single testing, it did not have a significant impact in the regression model. Our findings suggest that these factors independently contribute to a longer ICU length of stay in OSCC reconstruction surgeries. Furthermore, weak effect sizes were found in term of patients’ age, coronal artery disease, and albumin, whereas moderate correlations were identified in the COPD variables, BMI, tumor classification, and arterial hypertension. Parameters that were significant in single tests but not in the regression model were considered confounding variables [36]. However, the single tests did not provide a reliable correlation with the ICU length of stay, hence a regression model was used for further assessment.

In the multivariate regression model, several factors were significantly associated with a prolonged ICU length of stay. These factors include renal dysfunction, peripheral venous disease, postoperative complications and heart failure. Our study supports that these perioperative risk factors have a significant impact on the ICU length of stay following advanced oral squamous cell carcinoma surgery with free flap reconstruction.

Increased age of patients and longer operation times have been considered as risk factors for postoperative delirium and ICU length of stay [20, 21, 37]. Our results confirmed that after OSCC reconstructive surgery, increased tumor classification and patients age were significantly correlated with prolonged ICU length of stay. However, these parameters were found to contribute as confounding variables in combination with other factors, rather than being a single significant risk factor. This finding was similar to the results reported by Bhama et al.’s finding [19].

Previously, the impact of perioperative risk factors in ICU length of stay has been studied in various contexts, such as staffing effects, the impact of different sedation protocols, [9, 19] or prognostic implication of comorbidities, [29] and complication rates during ICU admission [38]. Studies that focus on ICU length of stay as the primary outcome have utilized scoring systems, such as the Acute Physiological and Chronic Health Evaluation (APACHEII) [39], the Charlson Comorbidity Index (CCI) [40], or the Simplified Physiological Score (SAPSII) [41], to investigate the relationship between perioperative parameters and ICU length of stay. However, these comorbidity indices have some limitations, as it is challenging to retrospectively identify and analyze a wide range of perioperative parameters into a single score. Furthermore, these indices are not often used in routine perioperative oral and maxillofacial assessments, but rather in anesthesiology to measure overall mortality or overall patient disease risk in ICU management. In contrast, this study focuses on perioperative parameters that are routinely assessed in every patient before oral and maxillofacial reconstructive surgeries, and evaluates which of them might serve as a reliable indicator for a longer ICU length of stay.

According to our results, in all identified parameters that were independent significantly correlated with ICU, the mean of ICU admission time was increased by 73.5% (three days or more). Notably, the highest correlation with ICU length of stay was found in renal dysfunction (GFR < 60 ml/ min), heart failure (class III), peripheral vascular disease and postoperative complications, which means these parameters were strongly correlated with longer ICU time. The fact that renal dysfunction can affect the body’s organ and recovery process by decreasing the elimination rate of toxin in the blood plasma, leading to impairment of cardio-pulmonary function. In general, unstable renal and cardio-pulmonary systems have critical consequences for the health condition, morbidity, and mortality [42].

The prolonged ICU length of stay negatively affects the patient’s health condition and increases 5-year mortality [10, 19]. Furthermore, 64,000 yuan is added as a charge for every 48 h of prolonged stay in the ICU. Similar findings were found in other published papers evaluating ICU hospital costs [9, 31]. This study has several limitations, such as being a retrospective single-center study, missing the evaluations of different flaps, and lacking data on certain surgical complications including the need for tracheostomy.

## Conclusion

Patient with renal dysfunction, peripheral vascular disease or high NYHA stages are at higher risk of experiencing a prolonged ICU length of stay To reduce the ICU length of stay and improve overall patient health outcomes, it is crucial to identify these risk factors early in patients scheduled for OSCC resection. In high-risk patients, providing appropriate and highly trained medical support and a suitable nursing ratio is of utmost importance compared to low-risk patients.

## Data Availability

The datasets used and/or analyzed during this study are available from the corresponding author on reasonable request.
